# The Effects of Body Mass Index on In-Hospital Mortality and Outcomes in Patients With Heart Failure: A Nationwide Analysis

**DOI:** 10.7759/cureus.22691

**Published:** 2022-02-28

**Authors:** Ahmed Brgdar, John Gharbin, Ayman Elawad, Sabah Khalafalla, Adey Bishaw, Abimbola F Balogun, Mohamed E Taha

**Affiliations:** 1 Internal Medicine, Howard University Hospital, Washington, DC, USA; 2 Cardiovascular Medicine, Howard University, Washington, DC, USA

**Keywords:** cardiovascular, heart failure, mortality, in-hospital, s: obesity paradox

## Abstract

Background: ​​​​​​Heart failure (HF) remains one of the leading causes of death in the United States. While many large-scale studies show a positive relationship between cardiovascular mortality and body mass index (BMI), several studies have also observed lower mortality rates among obese HF patients. Therefore, we sought to assess the impact of BMI on in-hospital outcomes in patients admitted with HF.

Methods: Patients hospitalized with congestive heart failure (CHF) diagnosis between 2005 and 2014 were identified from the US National Inpatient Sample database using the International Classification of Diseases, Ninth Revision, Clinical Modification diagnostic and procedural codes. The sample was divided into three groups based on their BMI. In-hospital outcomes were assessed in different groups and sub-groups.

Results: We identified 8,674,190 patients admitted with a primary diagnosis of HF, out of which 1.8% had BMI between 30 and 39.9 kg/m^2^ and 3.7% had BMI >40 kg/m^2^. In-hospital mortality was reported in 5.6% of patients with BMI <30 kg/m^2^, compared to 2.3% in those with BMI 30-39.9 kg/m^2^ and 3.1% in the group with BMI >40 kg/m^2^. After adjusting for various confounders, in-hospital mortality was lower in those with BMI 30-39.9 kg/m^2 ^than those with BMI <30 kg/m^2^ (OR 0.56; CI 0.51-0.62). Similarly, in-hospital mortality was lower in those with BMI >40 kg/m^2^ than those with BMI <30 (OR 0.87; CI 0.81-0.92).

Conclusion: Even though this study supports the findings of previous smaller studies illustrating the existence of the “obesity paradox” in HF hospitalizations, the pathogenesis behind this paradoxical effect is still unclear.

## Introduction

Heart failure (HF) is a growing health problem in the United States (US). The American Heart Association estimated a 46% increase in the prevalence of HF from 2012 to 2030 [1,2]. It is also a leading cause of death, accounting for one out of every nine deaths in the United States (US) [3].

Earlier studies have shown that the risk of HF increases with body mass index (BMI) and abdominal adiposity [4]. Several large-scale studies suggest that individuals in the obese BMI category may have a nearly two-fold increase in HF risk compared to the normal BMI population [5,6]. A follow-up analysis from the Framingham study demonstrated high BMI as an independent risk factor for developing HF, coronary artery disease (CAD), stroke, and overall cardiovascular disease (CVD) death [7]. The effects of obesity on CVD may be mediated indirectly through intervening risk factors, such as hypertension, dyslipidemia, and diabetes [8], or directly by altering the underlying morphological and functional hemodynamics [9].

Given this increased CVD risk, investigators may presume that obese individuals with HF would have higher mortality. Some studies, however, have shown this assumption to be incorrect [10]. In contrast to lean individuals, overweight and obese patients diagnosed with HF appear to have a better prognosis [11,12]. Additionally, earlier studies have suggested increased mortality rates and in-hospital complications in normal weight and lean patients undergoing coronary angiography compared to obese patients [13,14]. This paradoxical effect is known in the literature as the “obesity paradox” [15,16]. In addition to HF, the obesity paradox has been recognized in many chronic diseases such as cancers [17].

Some clinical studies have concluded that this relationship is causal, arguing that the state of overweight/obese is "protective" [18,19]. For example, the greater metabolic reserves in obese patients may confer protection against catabolic states such as HF, which can increase the risk of cachexia and death [20]. Indeed, a recent study showed that HF patients exhibit increased fat and muscle wasting and that higher fat mass (but not muscle mass) was independently associated with lower rates of readmissions or cardiac death [21]. These findings challenge the standard guidelines on weight management for many chronic illnesses.

It is noteworthy that most prior studies supporting the obesity paradox are epidemiological studies, and at least two sources of potential bias are likely to be important. First, there may be residual confounding, such as cigarette smoking, as smoking is inversely related to BMI and a key risk factor for mortality. For example, in the relationship between BMI and all-cause mortality in the general population, the obesity paradox is observed in current and former smokers, but not in non-smokers, making smoking an effect modifier [22]. The second is collider stratification, which is a specific selection bias that occurs when common causes of a disease and an outcome affect inclusion into the analysis [23].

Nevertheless, the obesity paradox has been repeatedly observed in several cardiovascular and cerebrovascular diseases, including intracerebral hemorrhage [24,25], peripheral arterial disease [26], acute myocardial infarction [27], and hypertension [28]. While all of these studies describe better survival outcomes in a nationally representative sample of in-hospital patients, especially among those who are mildly obese, the effect of obesity on in-hospital mortality outcomes in a nationally representative sample of HF patients has not been previously studied. Given this gap in the literature, we have investigated the trends of hospitalization and in-hospital outcomes associated with HF and BMI using the extensive US National Inpatient Sample (NIS) database.

## Materials and methods

Study design and patient population

The data for this population-based retrospective observational cohort study was obtained from the National Inpatient Sample (NIS) database [29]. The NIS database is sponsored by the Health Care Cost and Utilization Project (HCUP) and represents 95% of the hospitalizations, including in-patient admission from more than 1000 hospitals based in the United States. The NIS is a 20% stratified sample that is assessed every year for internal validity [30].

HF-related hospital admissions of individuals aged 18 years or above between 2005 and 2014 were identified from the NIS database using International Classification of Diseases, Ninth Revision, Clinical Modification (ICD-9-CM) discharge diagnosis codes in primary or secondary positions: 402.01, 402.11, 402.91, 404.01, 404.03, 404.11, 404.13, 404.91, 404.93, 428.00, 428.01, 428.09, 428.20, 428.21, 428.22, 428.23, 428.30, 428.31, 428.32, 428.33, 428.40, 428.41, 428.42, and 428.43 [31]. To identify HF with preserved ejection fraction (HFpEF), we included ICD-9-CM diagnostic codes 428.31 and 428.33 (acute diastolic heart failure) with the exclusion of ICD-9-CM diagnostic codes for systolic and diastolic combined heart failure codes. This method has been used previously [32].

To explore the relationship between obesity and HF, the study sample was divided into the following three groups according to BMI: group 1 comprised of those with BMI ≥40 kg/m^2^ (ICD-9-CM: V85.4), and group 2 comprised of individuals with BMI between 30 and 39 kg/m^2^ (ICD-9-CM: V85.3). The remaining hospitalizations were considered below BMI 30 kg/m^2 ^(group 3). Comorbidities were identified using the Elixhauser method [33], and Charlson’s comorbidity index (CCI) was used to describe the severity of comorbid conditions [34]. The CCI score ranges from 0-3, with higher scores representing the more significant burden of comorbid conditions.

Outcomes

The primary endpoints were trends of hospitalization and in-hospital mortality. The secondary endpoints included gender differences in mortality, the impact of obesity on various sub-groups with HF such as people with diabetes, HFpEF, and HF with reduced ejection fraction (HPrEF), or prior recipients of diagnostic catheterization, heart transplant, or left ventricular assist device (LVAD).

Statistical analysis

SAS 9.4 University Edition (Cary, NC: SAS Institute Inc.) was used for the analysis. Continuous data were represented as mean with standard deviation, while categorical data were represented in frequencies in percentage. Student’s t-test was utilized for continuous data and the chi-square test for categorical data. All tests were two-sided, and a p-value of <0.05 was considered statistically significant. Multivariate logistic regression analysis was performed to assess the effect of obesity and morbid obesity on in-hospital mortality. We analyzed three separate models for each sub-group. In the first model, age, gender, race, and median household income were included. In the second model, we included age, gender, race, median household income, and CCI. In the final model, we included age, gender, race, median household income, and Elixhauser comorbidities. We applied this model to several sub-groups (diabetic individuals, males, females, White population, African American population, HFpEF, and HFrEF) analysis.

## Results

A total of 8,674,190 patients were identified with a primary diagnosis of HF between 2005 and 2014, out of which 160,247 patients (1.8%) had a BMI between 30 and 39.9 kg/m^2^ while 320,367 patients (3.7%) had BMI >40 kg/m^2^ (Table 1). The average age of hospitalization was 73.1 (SD 13.9) years, with significant between group differences. The average age in the group with BMI below 30 kg/m^2^ was 73.6 years, while for patients with BMI between 30 and 39.9 kg/m^2^, the average age was 67.6 years, and that for the group with BMI >40 kg/m^2^ was 62 years. The overall sample comprised 52.7% males and 47.3% females; however, this distribution was unequal in the group with BMI >40 kg/m^2^ (61% males and 39% females).

**Table 1:** Baseline characteristics of heart failure hospitalizations by body mass index (BMI). Data are presented as mean (SD) or %.

Variables		Total	BMI (kg/m^2^)			p-Value
			<30	30-39.9	≥40	
Overall (n)		8,674,190	8,193,576	160,247	320,367	-
Age (years)		73.1 (13.9)	73.6 (13.7)	67.6 (12.7)	62.0 (12.4)	<0.001
Gender	Males	52.7%	52.4%	50.0%	61.0%	<0.001
	Females	47.3%	47.6%	50.0%	39.0%	
Race	Whites	72.4%	72.7%	68.1%	66.8%	<0.001
	Blacks	16.1%	15.7%	19.1%	23.9%	
	Hispanics	6.9%	6.9%	8.6%	6.1%	
	Asians	1.7%	1.8%	1.3%	0.7%	
	Others	2.9%	2.9%	2.9%	2.5%	
Median household income (percentile)	0-25th	32.0%	31.9%	32.2%	36.0%	<0.001
	26-50th	26.7%	26.6%	26.9%	28.1%	
	51-75th	22.8%	22.8%	23.5%	22.3%	
	76-100th	18.5%	18.7%	17.3%	13.6%	
Charlson’s comorbidity index	0	0.04%	0.04%	0.01%	0.02%	<0.001
	1	16.5%	16.8%	10.3%	10.7%	
	2	25.6%	25.8%	21.0%	22.2%	
	≥3	57.9%	57.3%	68.6%	67.0%	
Elixhauser comorbidity	Hypertension	65.6%	65.2%	76.0%	72.0%	<0.001
	Diabetes	30.8%	30%	43.3%	45.2%	<0.001
	Renal failure	30.0%	29.8%	34.9%	32.6%	<0.001
	Peripheral vascular disease	11.6%	11.7%	12.6%	8.3%	<0.001
	Liver disease	2.6%	2.6%	3.3%	3.5%	<0.001
	Pulmonary circulation disorders	5.1%	4.9%	6.7%	9.6%	<0.001
In-hospital mortality		5.4%	5.6%	2.3%	3.1%	<0.001
Length of stay		6.4 (7.5)	6.4 (7.5)	6.1 (6.1)	7.0 (7.8)	<0.001

While 16.1% of the patients in our overall sample were African Americans, they were disproportionately represented in the 30-39.9 kg/m^2^ (19.1%) >40 kg/m^2^ (23.9%) BMI categories. Overall, 58.7% of patients had a median household income below the 50th percentile; however, 64.1% of those with BMI >40 kg/m^2^ had a median household income below the 50th percentile. A significant burden of comorbidities was observed in HF hospitalizations, with 63.5% hospitalizations scoring ≥3 in the Charlson’s comorbidity index. Overall, in-hospital mortality was 5.4%, while in-hospital mortality was 2.3% in the group with BMI 30-39.9 kg/m^2^ and 3.1% in the group with BMI >40 kg/m^2^. The overall median length of stay was 6.4 days, while those with BMI >40 kg/m^2^ were seven days (Table 1).

After adjusting for various confounders, in-hospital mortality was lower in those with BMI 30-39.9 kg/m^2^ than those with BMI below 30 kg/m^2^ (OR 0.56; CI 0.51-0.62) (Table 2). Similarly, in-hospital mortality was lower in those with BMI >40 kg/m^2^ compared to those with BMI below 30 kg/m^2^ (OR 0.87; CI 0.81-0.92). Interestingly, hospitalizations in the morbid obesity group had lower odds ratio than obese in all sub-groups. In diabetic individuals, in-hospital mortality was lower when admitted with obesity than BMI below 30 kg/m^2^ (OR 0.60; CI 0.52-0.70). Also, in-hospital mortality was lower in morbidly obese diabetics than among those with BMI below 30 kg/m^2^ (OR 0.86; CI 0.78-0.95) (Table 2).

**Table 2:** Comparison of the overall in-hospital mortality and in patients with diabetes admitted primarily for heart failure between 2005 and 2014. CCI: Charlson’s comorbidity index; aOR: adjusted odds ratio; CI: confidence interval

	Total in-hospital mortality	p-Value	Diabetic patients	p-Value
BMI 30-39.9 kg/m^2^	n=1,038,092 aOR (CI 95%)		n=338,180 aOR (CI 95%)	
Model 1 (age, gender, race, median household income)	0.52 (0.47-0.58)	<0.001	0.59 (0.50-0.69)	<0.001
Model 2 (age, gender, race, median household income, CCI)	0.51 (0.46-0.57)	<0.001	0.58 (0.50-0.68)	<0.001
Model 3 (age, gender, race, median household income, Elixhauser comorbidity)	0.56 (0.51-0.62)	<0.001	0.60 (0.52-0.70)	<0.001
BMI ≥40 kg/m^2^	n=1,073,326 aOR (CI 95%)		n=354,616 aOR (CI 95%)	
Model 1 (age, gender, race, median household income)	0.79 (0.75-0.89)	<0.001	0.83 (0.75-0.92)	<0.001
Model 2 (age, gender, race, median household income, CCI)	0.78 (0.74-0.83)	<0.001	0.82 (0.74-0.91)	<0.001
Model 3 (age, gender, race, median household income, Elixhauser comorbidity)	0.87 (0.81-0.92)	<0.001	0.86 (0.78-0.95)	0.004

Obese males had a higher mortality rate (OR 0.52; CI 0.46-0.60) than obese females (OR 0.60; CI 0.52-0.69). Similarly, morbidly obese male hospitalizations were associated with higher mortality rates (OR 0.87; CI 0.79-0.96) than morbidly obese females (OR 0.89; CI 0.82-0.97). Furthermore, obese African Americans (OR 0.38; CI 0.28-0.52) had a higher in-hospital mortality rate than obese White patients (OR 0.57; CI 0.51-0.64). Similarly, morbidly obese African Americans had a higher rate of in-hospital mortality (OR 0.85; CI 0.73-0.98) compared to morbidly obese White patients (OR 0.89; CI 0.82-0.96) (Table 3). In all sub-group analyses, in-hospital mortality was higher in obese compared to morbidly obese (Tables 2-4).

**Table 3:** Adjusted odds ratio for in-hospital mortality: stratified by gender and race. CCI: Charlson’s comorbidity index; aOR: adjusted odds ratio; CI: confidence interval; AA: African American *Gender not included in the model. **Race not included in the model.

	Males*	p-Value	Females*	p-Value	Whites**	p-Value	AA**	p-Value
BMI 30-39.9 kg/m^2^	n=520,506 aOR (CI 95%)		n=517,542 aOR (CI 95%)		n=623,524 aOR (CI 95%)		n=192,938 aOR (CI 95%)	
Model 1 (age, gender, race, median household income)	0.48 (0.42-0.56)	<0.001	0.57 (0.50-0.66)	<0.001	0.54 (0.48-0.61)	<0.001	0.35 (0.26-0.49)	<0.001
Model 2 (age, gender, race, median household income, CCI)	0.48 (0.41-0.55)	<0.001	0.56 (0.48-0.64)	<0.001	0.53 (0.47-0.59)	<0.001	0.35 (0.25-0.48)	<0.001
Model 3 (age, gender, race, median household income, Elixhauser comorbidity)	0.52 (0.46-0.60)	<0.001	0.60 (0.52-0.69)	<0.001	0.57 (0.51-0.64)	<0.001	0.38 (0.28-0.52)	<0.001
BMI ≥40 kg/m^2^	n=532,321 aOR (CI 95%)		n=540,962 aOR (CI 95%)		n=642,428 aOR (CI 95%)		n=204,065 aOR (CI 95%)	
Model 1 (age, gender, race, median household income)	0.78 (0.71-0.86)	<0.001	0.84 (0.77-0.91)	<0.001	0.83 (0.77-0.89)	<0.001	0.76 (0.66-0.89)	<0.001
Model 2 (age, gender, race, median household income, CCI)	0.77 (0.70-0.85)	<0.001	0.82 (0.76-0.89)	<0.001	0.81 (0.75-0.87)	<0.001	0.75 (0.65-0.87)	<0.001
Model 3 (age, gender, race, median household income, Elixhauser comorbidity)	0.87 (0.79-0.96)	0.004	0.89 (0.82-0.97)	0.008	0.89 (0.82-0.96)	0.002	0.85 (0.73-0.98)	0.030

Obese hospitalizations with HFpEF had higher in-hospital mortality (OR 0.53; CI 0.44-0.63) than obese hospitalization with HFrEF (OR 0.62; CI 0.53-0.72). However, no difference was noted between morbidly obese patients with HFpEF (OR 0.92; CI 0.83-1.01) and morbidly obese patients with HFrEF (OR 0.93, CI 0.83-1.03) (Table 4).

**Table 4:** Adjusted Odds Ratio for in-hospital mortality in patients with HFpEF and HFrEF. CCI: Charlson’s comorbidity index; aOR: adjusted odds ratio; CI: confidence interval

	HFpEF	p-Value	HFrEF	p-Value
BMI 30-39.9 kg/m^2^	n=307,546, aOR (CI 95%)		n=454,650, aOR (CI 95%)	
Model 1 (age, gender, race, median household income)	0.51 (0.43-0.61)	<0.001	0.58 (0.50-0.68)	<0.001
Model 2 (age, gender, race, median household income, CCI)	0.50 (0.42-0.59)	<0.001	0.57 (0.49-0.65)	<0.001
Model 3 (age, gender, race, median household income, Elixhauser comorbidity)	0.53 (0.44-0.63)	<0.001	0.62 (0.53-0.72)	<0.001
BMI ≥40 kg/m^2^	n=326,876, aOR (CI 95%)		n=463,155, aOR (CI 95%)	
Model 1 (age, gender, race, median household income)	0.86 (0.78-0.95)	0.003	0.86 (0.77-0.95)	0.003
Model 2 (age, gender, race, median household income, CCI)	0.86 (0.78-0.94)	0.002	0.84 (0.75-0.93)	<0.001
Model 3 (age, gender, race, median household income, Elixhauser comorbidity)	0.92 (0.83-1.01)	0.08	0.93 (0.84-1.03)	0.18

In terms of clinical management, diagnostic and therapeutic angiography utilization was higher among those with BMI 30-39.9 kg/m^2^ (11.4%), compared to those in BMI less than 30 kg/m^2^ (7.6%) and BMI >40 kg/m^2^ (7.3%) groups (Table 5). Similarly, heart transplantation was utilized in 0.05% in those with BMI 30-39.9 kg/m^2^, compared to 0.04% in those with BMI less than 30 kg/m^2^. LVAD utilization rate was also higher among those with BMI 30-39.9 kg/m^2^ (0.08%) compared to 0.05% in the other BMI groups (Table 5).

**Table 5:** Angiography, heart transplantation, and left ventricular assist device (LVAD) utilization among patients admitted with heart failure stratified by body mass index (BMI).

Variables	Total	BMI (kg/m^2^)			p-Value
		<30	30-39.9	>40	
Overall (n)	8,674,190	8,193,576	160,247	320,367	-
Angiography (%)	7.7%	7.6%	11.4%	7.3%	<0.001
Heart transplantation (%)	0.04%	0.04%	0.05%	0.0%	<0.001
LVAD (%)	0.05%	0.05%	0.08%	0.05%	<0.001

## Discussion

This study demonstrates the impact of obesity on HF hospitalizations from the largest all-payer database in the US and supports the findings of previous smaller studies illustrating the existence of the “obesity paradox” in HF hospitalizations. The increased mortality risk in non-obese patients was independent of other risk factors such as diabetics, gender, race, HFpEF, and HFrEF. Notably, morbidly obese patients were at a higher risk compared to obese patients in all sub-groups. Additionally, males and African Americans were at a higher risk of in-hospital mortality than females and Caucasians, respectively. Furthermore, HFpEF hospitalizations had a higher incidence of mortality than HFrEF in an adjusted model in this study. Together our findings suggest that the protective effect from the “obesity paradox” may be seen less in morbidly obese HF hospitalizations than obese patients.

Our study is in line with an earlier analysis of HF patients in the 2013-2014 Nationwide Readmission Database (NRD) in terms of overall mortality trends with some insightful differences [35]. While we observed in-hospital mortality rates of 5.6%, 2.3%, and 3.1% among non-obese, obese, and morbidly obese HF patients, respectively, Gajulapalli et al. reported lower mortality rates of 3.1% and 1.8% among rehospitalized non-obese and obese HF patients, respectively [35]. It is plausible that HF patients requiring readmission generally have a lower mortality risk. For instance, in an analysis of 2010-2014 NRD, the in-hospital mortality rate of patients with ST‐segment-elevation myocardial infarction (STEMI) was 8.7% during index admission but only 4.6% during readmission [36]. However, the higher mortality rates observed in our study could also be attributable to differences in the design of NIS and NRD or the timeframe of the analysis (2013-2014 vs. 2004-2014).

The term “obesity paradox” has been described previously, suggesting lower mortality in obese patients [18,19]. This association may be derived from lower hospitalization age in obese and morbidly obese patients, as seen in this study. Moreover, higher comorbidities noticed in obese and morbidly obese patients may have a negative effect on the pathogenesis of HF. Additionally, the symptoms and signs of HF could present earlier in obese patients [37]. Furthermore, the cytokine, endocrine, and neuroendocrine profile of obese patients may confer a survival advantage [20]. For instance, Mehra et al. observed significantly lower B-type natriuretic peptide (BNP) among obese HF patients [38], which may potentially lead to patients being symptomatic at an early stage of HF and, in turn, to early treatment [20].

Concurring with these hypotheses, we observed higher rates of utilization of angiography, heart transplantation, and LVAD implantation among obese individuals compared to those with BMI <30 kg/m^2^ and morbidly obese individuals. These observations suggest that early diagnosis and treatment of HF in the obese group may produce a lead-time bias. Also, obese patients may be more likely to be diagnosed with HF given their tendency to develop peripheral edema, dyspnea, or easy fatigability, symptoms that could be entirely non-cardiogenic in origin, leading to misclassification bias.

As noted in previous studies, we observed that females had a survival advantage compared to males [39]. Several hypotheses have been proposed for this advantage in line with the possible mechanisms of the obesity paradox discussed above, including estrogen effect, higher adiposity and associated favorable survival, and high fatty acid turnover in females [40-42]. However, these association effects may be observed less in those hospitalizations with morbidly obese as this study observed higher mortality in morbidly obese patients than obese patients. Although we observed a slightly higher mortality risk in morbidly obese patients, this risk was still lower than normal-weight hospitalizations.

There is, however, inconsistency on the impact of obesity on the ejection fraction in HF patients. In one study in postmenopausal women, HFpEF was strongly associated with obesity [43], while another study suggests that obesity is an independent risk factor for HFpEF [44]. A recent meta-analytic review showed that the obesity paradox in HF, specially HFrEF, was evident in patients older than 75 years or with at least one comorbidity but not among younger patients or those with only HF [45]. Although we observed a significantly lower mortality risk in obese patients with either HFpEF or HFrEF, the effect was weaker in the morbidly obese group. Notably, the patients in our morbidly obese groups were significantly younger (62 years vs. 73.1 years overall) and had lower Charlson’s comorbidity index score than those in obese groups. Therefore, it is likely that other underlying factors such as higher rates of glucose intolerance, metabolic syndrome, or chronic inflammation, all of which are known to worsen cardiovascular outcomes and mortality, may diminish the survivability advantage in morbidly obese patients with HFpEF or HFrEF compared to obese patients [46].

Limitations

Several limitations are present that may affect the outcome of this study. There is a potential for inherent biases in any retrospective study where we may not be able to fully adjust for residual or unmeasured confounding variables, which, in turn, may affect our estimates for the reported association. Additionally, the results of our analysis can only be viewed as associational and hypothesis-generating with no ability to detect causation. There was also a paucity of data on pre- or in-hospital pharmacotherapy for HF in the NIS database, which precluded further analysis and adjustment regarding the impact of prevalent treatment of HF on outcomes. Data on pre-hospital left ventricular function and the New York Heart Association (NYHA) functional class was also limited, affecting the stratification of these crucial measures.

Moreover, the prevalence of obesity (1.8%) and morbid obesity (3.7%) in our study population is much lower than national estimates [47]. However, most patient sub-populations in the NIS have lower obesity prevalence than national estimates: between 2011 and 2014, obesity diagnosis among in-patients in the NIS ranged between 11.1% and 13.7% [48]. Among patients hospitalized with intracerebral hemorrhage during 2007-2014, the prevalence of obesity and morbid obesity was even lower at 4.86% and 2.15%, respectively [24]. Similarly, only 14.7% of patients hospitalized due to a primary diagnosis of pulmonary arterial hypertension between 2003 and 2011 were obese [28]. While administrative databases such as the NIS may have the risk of under coding health indicators, direct surveys such as the Behavioral Risk Factor Surveillance System used for national estimates have other limitations due to their reliance on self-reporting [49]. Therefore, findings from any database study must be seen in light of their associated limitations.

## Conclusions

Even though this study supports the findings of previous smaller studies illustrating the existence of the “obesity paradox” in CHF hospitalizations, the pathogenesis behind this paradoxical effect and the specifics that may have contributed to the observed reduction in mortality remain unclear. Therefore, more studies, preferably large prospective trials, are needed to clarify this association, especially considering the globally rising prevalence of obesity.

## References

[1] Heidenreich PA, Albert NM, Allen LA (2013). Forecasting the impact of heart failure in the United States: a policy statement from the American Heart Association. Circ Heart Fail.

[2] Benjamin EJ, Blaha MJ, Chiuve SE (2017). Heart disease and stroke statistics-2017 update: a report from the American Heart Association. Circulation.

[3] Mozaffarian D, Benjamin EJ, Go AS (2016). Heart disease and stroke statistics-2016 update: a report from the American Heart Association. Circulation.

[4] Aune D, Sen A, Norat T, Janszky I, Romundstad P, Tonstad S, Vatten LJ (2016). Body mass index, abdominal fatness, and heart failure incidence and mortality: a systematic review and dose-response meta-analysis of prospective studies. Circulation.

[5] Kenchaiah S, Evans JC, Levy D (2002). Obesity and the risk of heart failure. N Engl J Med.

[6] Wilson PW, D'Agostino RB, Sullivan L, Parise H, Kannel WB (2002). Overweight and obesity as determinants of cardiovascular risk: the Framingham experience. Arch Intern Med.

[7] Hubert HB, Feinleib M, McNamara PM, Castelli WP (1983). Obesity as an independent risk factor for cardiovascular disease: a 26-year follow-up of participants in the Framingham Heart Study. Circulation.

[8] Valavanis IK, Mougiakakou SG, Grimaldi KA, Nikita KS (2010). A multifactorial analysis of obesity as CVD risk factor: use of neural network based methods in a nutrigenetics context. BMC Bioinformatics.

[9] Morricone L, Malavazos AE, Coman C, Donati C, Hassan T, Caviezel F (2002). Echocardiographic abnormalities in normotensive obese patients: relationship with visceral fat. Obes Res.

[10] Oga EA, Eseyin OR (2016). The obesity paradox and heart failure: a systematic review of a decade of evidence. J Obes.

[11] Horwich TB, Fonarow GC, Hamilton MA, MacLellan WR, Woo MA, Tillisch JH (2001). The relationship between obesity and mortality in patients with heart failure. J Am Coll Cardiol.

[12] Fonarow GC, Srikanthan P, Costanzo MR, Cintron GB, Lopatin M (2007). An obesity paradox in acute heart failure: analysis of body mass index and inhospital mortality for 108,927 patients in the Acute Decompensated Heart Failure National Registry. Am Heart J.

[13] Gruberg L, Weissman NJ, Waksman R (2002). The impact of obesity on the short-term and long-term outcomes after percutaneous coronary intervention: the obesity paradox?. J Am Coll Cardiol.

[14] Gurm HS, Whitlow PL, Kip KE (2002). The impact of body mass index on short- and long-term outcomes inpatients undergoing coronary revascularization. Insights from the bypass angioplasty revascularization investigation (BARI). J Am Coll Cardiol.

[15] Hamzeh N, Ghadimi F, Farzaneh R, Hosseini SK (2017). Obesity, heart failure, and obesity paradox. J Tehran Heart Cent.

[16] Shah R, Gayat E, Januzzi JL Jr (2014). Body mass index and mortality in acutely decompensated heart failure across the world: a global obesity paradox. J Am Coll Cardiol.

[17] Lennon H, Sperrin M, Badrick E, Renehan AG (2016). The obesity paradox in cancer: a review. Curr Oncol Rep.

[18] Veronese N, Cereda E, Solmi M (2015). Inverse relationship between body mass index and mortality in older nursing home residents: a meta-analysis of 19,538 elderly subjects. Obes Rev.

[19] Doehner W, von Haehling S, Anker SD (2015). Protective overweight in cardiovascular disease: moving from 'paradox' to 'paradigm'. Eur Heart J.

[20] Lavie CJ, Alpert MA, Arena R, Mehra MR, Milani RV, Ventura HO (2013). Impact of obesity and the obesity paradox on prevalence and prognosis in heart failure. JACC Heart Fail.

[21] Ohori K, Yano T, Katano S (2021). High percent body fat mass predicts lower risk of cardiac events in patients with heart failure: an explanation of the obesity paradox. BMC Geriatr.

[22] Aune D, Sen A, Prasad M (2016). BMI and all cause mortality: systematic review and non-linear dose-response meta-analysis of 230 cohort studies with 3.74 million deaths among 30.3 million participants. BMJ.

[23] Hernán MA, Hernández-Díaz S, Robins JM (2004). A structural approach to selection bias. Epidemiology.

[24] Persaud SR, Lieber AC, Donath E (2019). Obesity paradox in intracerebral hemorrhage. Stroke.

[25] Javalkar V, Kuybu O, Davis D, Kelley RE (2020). Factors associated with inpatient mortality after intracerebral hemorrhage: updated information from the United States nationwide inpatient sample. J Stroke Cerebrovasc Dis.

[26] Ludhwani D, Wu J (2019). Obesity paradox in peripheral arterial disease: results of a propensity match analysis from the National Inpatient Sample. Cureus.

[27] Velazquez G, Gomez TM, Asemota I, Akuna E, Ojemolon PE, Eseaton P (2020). Obesity impacts mortality and rate of revascularizations among patients with acute myocardial infarction: an analysis of the National Inpatient Sample. Cureus.

[28] Agarwal M, Agrawal S, Garg L, Lavie CJ (2017). Relation between obesity and survival in patients hospitalized for pulmonary arterial hypertension (from a Nationwide Inpatient Sample Database 2003 to 2011). Am J Cardiol.

[29] (2021). Overview of the National (nationwide) Inpatient Sample (NIS). http://www.hcup-us.ahrq.gov/nisoverview.jsp.

[30] Doshi R, Rao G, Shlofmitz E, Donnelly J, Meraj P (2017). Comparison of in-hospital outcomes after percutaneous revascularization for peripheral arterial disease in patients with a body mass index of >30 kg/m2 versus ≤30 kg/m2 (from the National Inpatient Sample). Am J Cardiol.

[31] Bonow RO, Bennett S, Casey DE Jr (2005). ACC/AHA clinical performance measures for adults with chronic heart failure: a report of the American College of Cardiology/American Heart Association Task Force on performance measures (Writing Committee to Develop Heart Failure Clinical Performance Measures) endorsed by the Heart Failure Society of America. J Am Coll Cardiol.

[32] Goyal P, Almarzooq ZI, Horn EM (2016). Characteristics of hospitalizations for heart failure with preserved ejection fraction. Am J Med.

[33] Elixhauser A, Steiner C, Harris DR, Coffey RM (1998). Comorbidity measures for use with administrative data. Med Care.

[34] Austin SR, Wong YN, Uzzo RG, Beck JR, Egleston BL (2015). Why summary comorbidity measures such as the Charlson comorbidity index and Elixhauser score work. Med Care.

[35] Gajulapalli RD, Kadri A, Gad M, Chahine J, Nusairat L, Rader F (2020). Impact of obesity in hospitalized patients with heart failure: a nationwide cohort study. South Med J.

[36] Kim LK, Yeo I, Cheung JW (2018). Thirty-day readmission rates, timing, causes, and costs after ST-segment-elevation myocardial infarction in the United States: a national readmission database analysis 2010-2014. J Am Heart Assoc.

[37] Khalid U, Ather S, Bavishi C (2014). Pre-morbid body mass index and mortality after incident heart failure: the ARIC Study. J Am Coll Cardiol.

[38] Mehra MR, Uber PA, Park MH, Scott RL, Ventura HO, Harris BC, Frohlich ED (2004). Obesity and suppressed B-type natriuretic peptide levels in heart failure. J Am Coll Cardiol.

[39] Vest AR, Wu Y, Hachamovitch R, Young JB, Cho L (2015). The heart failure overweight/obesity survival paradox: the missing sex link. JACC Heart Fail.

[40] Herrero P, Soto PF, Dence CS, Kisrieva-Ware Z, Delano DA, Peterson LR, Gropler RJ (2005). Impact of hormone replacement on myocardial fatty acid metabolism: potential role of estrogen. J Nucl Cardiol.

[41] Mittendorfer B (2005). Sexual dimorphism in human lipid metabolism. J Nutr.

[42] Lavie CJ, Osman AF, Milani RV, Mehra MR (2003). Body composition and prognosis in chronic systolic heart failure: the obesity paradox. Am J Cardiol.

[43] Eaton CB, Pettinger M, Rossouw J (2016). Risk factors for incident hospitalized heart failure with preserved versus reduced ejection fraction in a multiracial cohort of postmenopausal women. Circ Heart Fail.

[44] Lam CS, Carson PE, Anand IS (2012). Sex differences in clinical characteristics and outcomes in elderly patients with heart failure and preserved ejection fraction: the irbesartan in heart failure with preserved ejection fraction (I-PRESERVE) trial. Circ Heart Fail.

[45] Marcks N, Aimo A, Januzzi JL Jr (2021). Re-appraisal of the obesity paradox in heart failure: a meta-analysis of individual data. Clin Res Cardiol.

[46] Kaur J (2014). A comprehensive review on metabolic syndrome. Cardiol Res Pract.

[47] Wang Y, Beydoun MA, Min J, Xue H, Kaminsky LA, Cheskin LJ (2020). Has the prevalence of overweight, obesity and central obesity levelled off in the United States? Trends, patterns, disparities, and future projections for the obesity epidemic. Int J Epidemiol.

[48] Thapa JR, Ingels JB, Chung SR, Thapa K, Chen Z, Zhang D (2020). In-patient obesity diagnosis, use of surgical treatment and associated costs by payer type in the United States: analysis of the National Inpatient Sample, 2011 through 2014. Clin Obes.

[49] Al Kazzi ES, Lau B, Li T, Schneider EB, Makary MA, Hutfless S (2015). Differences in the prevalence of obesity, smoking and alcohol in the United States Nationwide Inpatient Sample and the behavioral risk factor surveillance system. PLoS One.

